# Correlation Study of Squash Smear With Histopathology and Radiological Diagnosis of Space-Occupying Lesions of Brain and Spinal Cord

**DOI:** 10.7759/cureus.88400

**Published:** 2025-07-21

**Authors:** Amresh Kumar, Anju Singh, Richa Sharma, Mamta Kumari, Samrendra Kumar Singh

**Affiliations:** 1 Department of Pathology, Indira Gandhi Institute of Medical Sciences, Patna, IND; 2 Department of Neurosurgery, Indira Gandhi Institute of Medical Sciences, Patna, IND

**Keywords:** central nervous system, glial tumors, histopathology, radiological diagnosis, squash smear cytology

## Abstract

Background: The precise evaluation of tissue samples is crucial for diagnosing and managing central nervous system (CNS) tumors. Squash smear cytology offers a rapid intraoperative pathological assessment of space-occupying lesions in the nervous system. This study aims to evaluate the validity of intraoperative squash smear cytology for CNS tumors and compare its findings with histopathological and radiological diagnoses.

Material and methods: This prospective hospital-based observational study was conducted in the Department of Pathology in association with the Departments of Neurosurgery and Radiodiagnosis, from October 2022 to July 2024. Fifty patients with neurological symptoms, such as headaches, vomiting, seizures, and paralysis, who were diagnosed radiologically with intracranial or intraspinal space-occupying lesions and underwent surgery, were included. Institutional ethical approval was obtained, and preoperative radiological examinations (CT scan or MRI) were recorded. Squash smear cytology was performed using 100% methanol-fixed smears stained with rapid hematoxylin and eosin (H&E). The cytological diagnosis was correlated with histopathological examination, which was used for definitive diagnosis and comparison.

Results: The study included 50 patients aged 10 to 78 years (median age: 41 years), with 32 males (64%) and 18 females (36%). The most common radiological diagnosis was glioma (20, 40%), followed by meningioma (15, 30%). The frontal lobe was the most common tumor site (18, 36%), followed by the spinal cord (7, 14%). Squash smear cytology diagnosed gliomas (20, 40%), meningiomas (17, 34%), and brain metastases (2, 4%). A statistically significant correlation (p = 0.001) was found between squash smear and histopathological diagnosis, highlighting its accuracy.

Conclusion: Squash smear cytology is a sensitive, specific, rapid, and cost-effective modality for diagnosing CNS lesions. It requires minimal tissue and is valuable in settings where frozen section facilities are unavailable. When performed by an experienced pathologist, it serves as a reliable intraoperative diagnostic tool, aiding neurosurgeons in decision-making and ensuring effective patient management.

## Introduction

Evaluation of tissue samples is necessary for the treatment and management of central nervous system (CNS) tumors. Intracranial tumors have an annual incidence of 10 to 17 cases per 100,000 individuals, and intraspinal tumors one to two cases per 100,000 individuals [1]. Glial tumors constitute one of the most common categories of intracranial neoplasms, accounting for approximately 40.3% of all brain tumors [2]. A wide range of tumors, such as meningioma, schwannoma, small round blue cell tumors, lipomas, epidermoid cysts, hemangiomas, and metastases, are non-glial lesions. Because these non-glial tumors are relatively uncommon, knowledge and expertise in smear studies of these cases have not been well explored. When the biopsy sample received is small, arriving at a diagnosis is more difficult.

Therefore, accurate identification of the location, size, histological type, and grade of CNS tumors is needed for the optimal management of patients. Modern radiological techniques are excellent at determining the spatial boundaries of the tumor, but pathologists still must characterize these lesions exactly. Open craniotomy or stereotactic biopsy determines the amount of tissue available to pathologists. The key goal of intraoperative cyto-diagnosis in stereotactic biopsies is to verify the adequacy of the tissue sample, a task that was accomplished by many medical centers, as shown by various studies [3]. An intraoperative diagnosis allows the collection of additional tissue for ancillary techniques to help diagnose infections and lymphomas for open biopsies. It also helps surgeons to decide the extent of resection and to start postoperative adjuvant therapy. 

The squash smear technique is the most commonly used cytological method for intraoperative neuropathological examination. This technique was introduced by Eisenhardt and Cushing in the early 1930s [4] and is a simple, rapid, inexpensive, and accurate intraoperative diagnostic modality [5]. The squash smear technique is useful because it requires only small amounts of tissue for diagnosis, especially when resecting tissue from functionally critical areas of the brain [6]. As such, squash cytology is an important first-line tool for neurosurgeons in their decision-making. The smear is relatively easy to make, and standard staining protocols can be used. Squash cytology was considered to be a mirror image of histopathology [7-9]. However, for an accurate diagnosis, a detailed understanding of the smears from different CNS lesions is required.

Optimization of neurosurgical procedures requires intraoperative histological consultation, which refers to the rapid evaluation of tissue samples during surgery to aid in real-time clinical decision-making. Cytology and frozen section are both important diagnostic tools in this context, as they help prevent unnecessary extensive craniotomies, assist the neurosurgeon in accurately targeting the lesion during resection, and may influence the extent and approach of the surgery [10-12]. 

In developing countries such as India, where frozen section machines are limited and their maintenance is difficult, cytology is preferred to frozen section preparation for intraoperative diagnosis of CNS tumors. CNS tissue is softer and is better suited for squash cytology, but is a hindrance for frozen sections. Furthermore, brain tissue is friable, and therefore frozen sections can produce ice crystal artifacts that interfere with diagnosis. The purpose of this study is to describe our experience with squash cytology and discuss the possible diagnostic pitfalls.

The objective of the study was to evaluate the validity of rapid intraoperative diagnosis of CNS tumors using squash smear cytology by comparing its findings with histopathological and radiological diagnoses.

## Materials and methods

Study design

The study was designed as a prospective, hospital-based observational investigation. 

Source of study

The research was done in the Department of Pathology in association with the Departments of Neurosurgery and Radiodiagnosis from October 2022 to July 2024. Patients with symptoms like headache, vomiting, seizures, paralysis, or other neurological signs who were diagnosed radiologically with intracranial or intraspinal space-occupying lesions (SOLs) and underwent surgery were studied. For the study, 50 patients enrolled at IGIMS, Patna, were selected. The Institutional Research and Ethics Committee had approved that study before it began. All patients included in the study were informed about the objectives of the study, and written consent was taken. Before surgery, radiological examinations, such as CT scans or MRIs, were performed, and the results were duly recorded.

Inclusion and exclusion criteria

The study included all ages of patients who had space-occupying lesions (SOLs) in the brain and spinal cord and underwent surgery. Also included were cases with recurrent tumors after surgery for SOL in the brain and spinal cord. The patients who had symptoms like headache, vomiting, seizures, paralysis, or any other neurological signs and were diagnosed radiologically with intracranial or intraspinal space-occupying lesions were included in the study. Exclusion criteria included cases with extensive tumor necrosis without sufficient viable tumor cells, cases in which inadequate tissue was received intraoperatively, cases that proved to be inflammatory, and cases where histopathological evaluation could not be performed.

Method of squash smear preparation and examination

Subsequent surgery was performed on these patients, and tissue samples were immediately transported from the operating theater in normal saline, along with request forms containing relevant clinical and radiological data. Necrosis and hemorrhage were carefully inspected in the tissue samples. Areas that appeared grossly distinct were selected for smear preparation. Two or more tissue fragments, each approximately 2 mm in diameter, were chosen. A labeled glass slide was placed on the tissue fragment, and a second labeled slide was gently pressed on top without applying excessive pressure. Half of the prepared smears were fixed in 100% methanol and stained with rapid hematoxylin and eosin (H&E), while the remaining smears were stained using rapid Leishman's stain.

An intraoperative diagnosis was aimed to be provided within a target turnaround time of 30 minutes. All cytological observations were recorded and later compared with the corresponding histopathological diagnosis, which served as the gold standard. The cytological and histopathological evaluations were performed by two senior pathologists, each with over 10 years of experience in neuropathology. The same pathologist who performed the intraoperative cytological assessment also conducted the final histopathological evaluation for each respective case, ensuring consistency in diagnostic correlation.

Methods of statistical analysis

Categorical variables were described as frequencies with percentages. The diagnostic accuracy of the squash smear test was evaluated by calculating its sensitivity, specificity, positive predictive value (PPV), and negative predictive value (NPV), using histopathological examination (HPE) as the gold standard. Statistical significance was considered to be a p-value less than 0.05. SPSS software (version 26.0, IBM Corp., Armonk, NY) and Epi Info software (version 7.2.5, Centers for Disease Control and Prevention, Atlanta, GA, USA) were used for data classification. During data analysis, statistical tests such as the Chi-square test and Fisher's exact test were applied to evaluate associations between categorical variables. Sensitivity, specificity, PPV, and NPV were calculated to assess the diagnostic performance of squash smear cytology using histopathological findings as the gold standard.

## Results

A total of 50 patients with radiologically diagnosed SOLs of the brain or spinal cord were included in this study. The patients ranged in age from 10 to 78 years, with a median age of 41 years. Most cases occurred in the 41-50 year age group (26%), followed by the 31-40 year group (22%). Males accounted for 64% of cases (n = 32), while females made up 36% (n = 18). 

Radiological findings and lesion distribution

Gliomas were the most common radiological diagnosis, present in 40% of patients, followed by meningiomas (30%), schwannomas (8%), and metastatic lesions (4%). Less frequent diagnoses included hemangiomas, epidermoid cysts, lipomas, and medulloblastomas. Gliomas were the most frequent (40%), followed by meningiomas (30%) and schwannomas (8%). Other less common findings included metastasis, hemangioma, medulloblastoma, neurofibroma, vascular malformation, mesenchymal tumor, epidermoid cyst, and lipoma.

The frontal lobe was the most commonly affected region, involved in 36% of cases. Spinal cord lesions accounted for 14%, with the cerebellopontine angle (10%), cerebellum (8%), and other intracranial regions making up the remainder. The frontal lobe was the most commonly involved site (36%), followed by the spinal cord (14%) and the cerebellopontine angle (10%). Other locations included the cerebellum, ventricles, and base of skull.

Cytological evaluation via squash smear

Squash smear cytology yielded a diagnosis of glioma in 20 cases (40%), meningioma in 16 (32%), and schwannoma in 6 (12%). Other lesions identified included metastatic carcinoma (two cases), hemangioma (two cases), epidermoid cyst (one case), lipoma (one case), small round cell tumor (one case), and a spindle cell lesion (one case). Gliomas were most frequently diagnosed (40%), followed by meningiomas (32%) and schwannomas (12%). Other diagnoses included metastasis, hemangioma, epidermoid cyst, lipoma, spindle cell lesion, and small round cell tumor.

**Figure 1:**
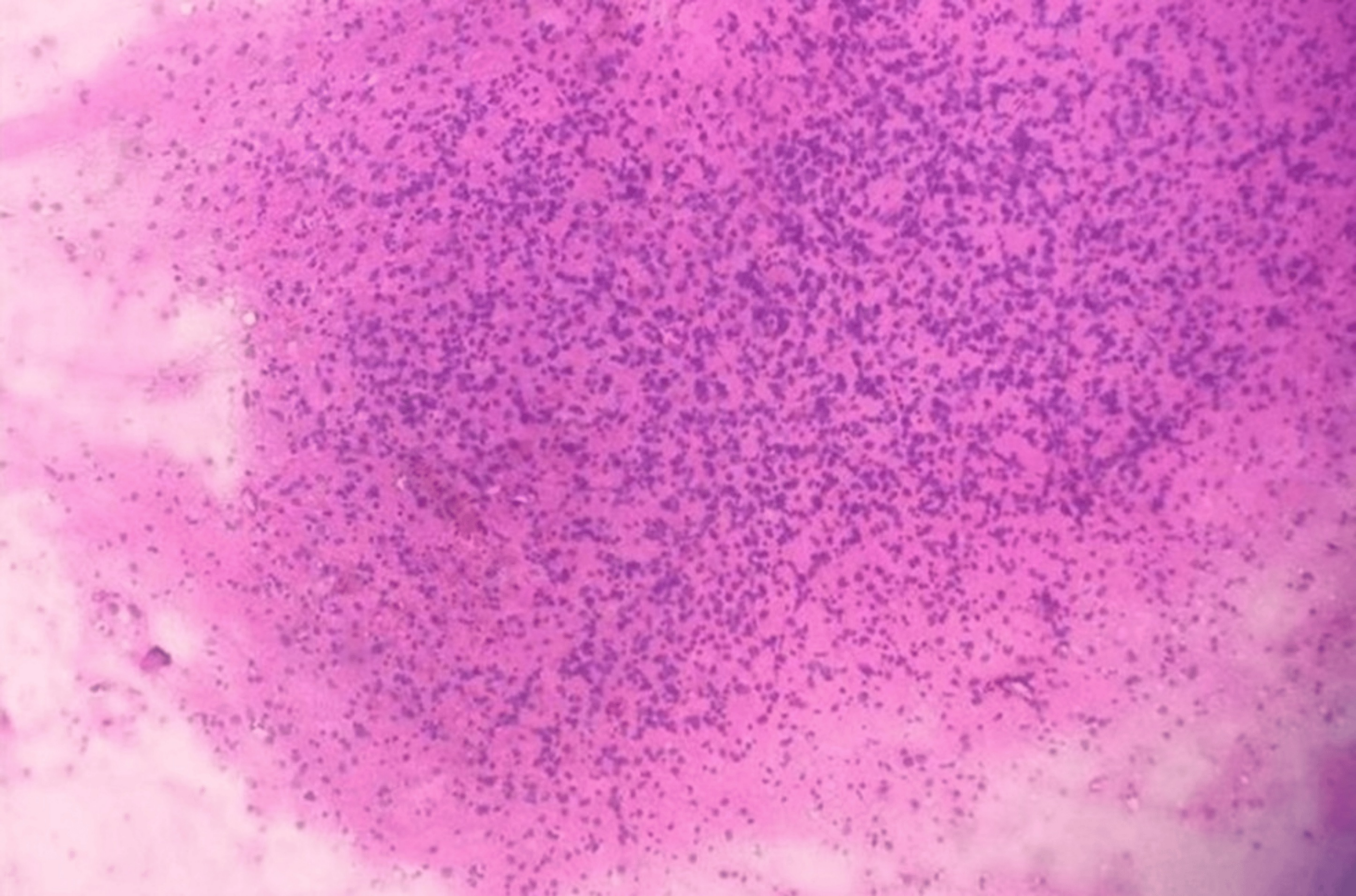
Low-grade glioma in squash cytology (10x).

The smears were fixed in 100% methanol for one to two minutes and stained using the rapid hematoxylin and eosin (H&E) method, which involved brief immersion in hematoxylin followed by eosin, rinsing, air drying, and mounting with distyrene, plasticizer, and xylene (DPX). Low-grade gliomas demonstrated moderate cellularity with a fibrillary background and uniform nuclei. Figure 1 shows a low-grade glioma on squash cytology (10x magnification), highlighting these cytological features.

**Figure 2:**
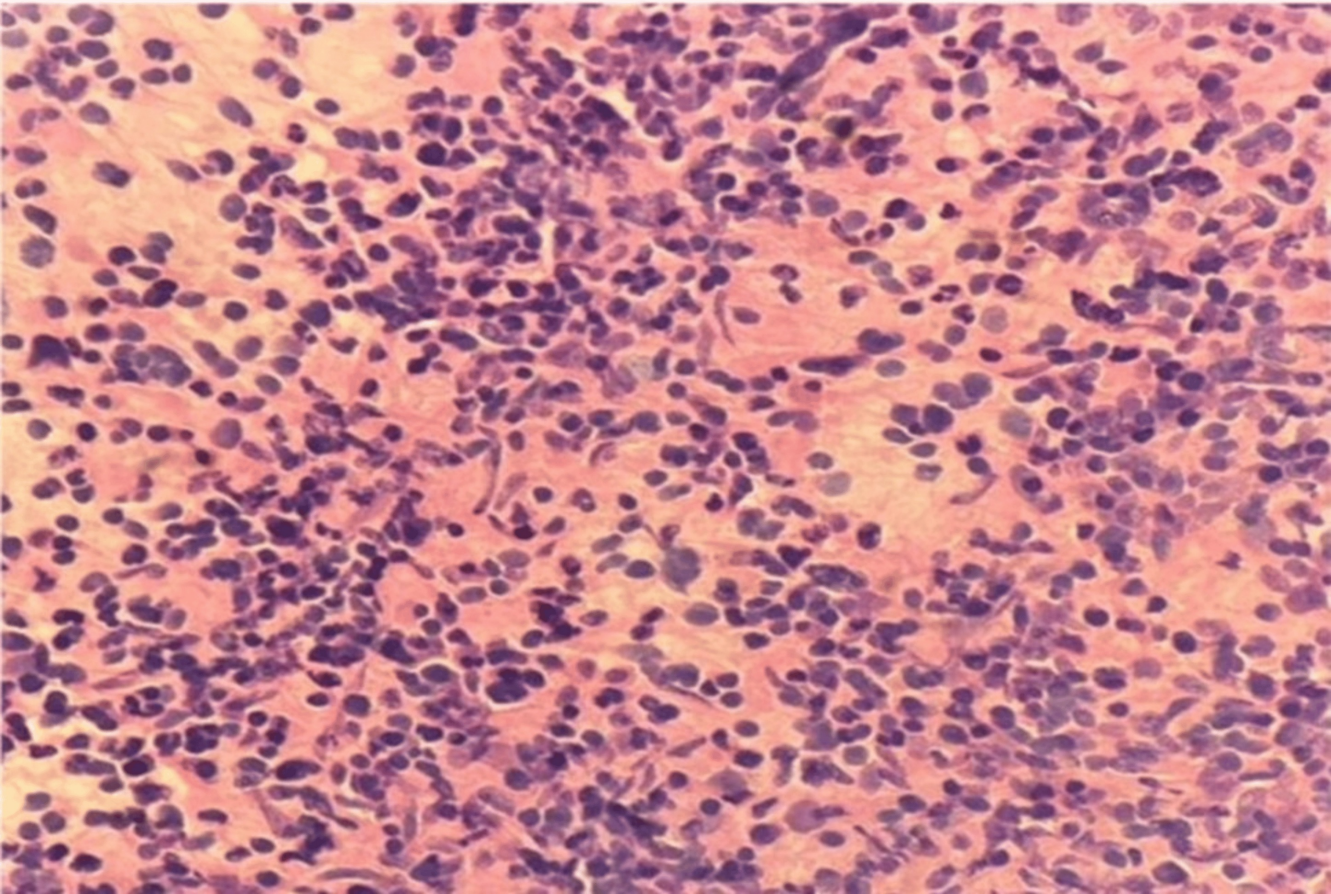
Glioblastoma in squash cytology (40x).

High-grade gliomas, particularly glioblastoma multiforme, were characterized by marked nuclear pleomorphism, brisk mitotic activity, and areas of necrosis on squash cytology. These cytological features are well demonstrated in Figure 2, captured under 40x magnification. The smear was fixed in 100% methanol for one to two minutes and stained using the rapid hematoxylin and eosin (H&E) technique, which involved brief immersion in hematoxylin followed by eosin, rinsing in water, air drying, and mounting with DPX. This method enabled crisp visualization of the diagnostic cellular architecture.

**Figure 3:**
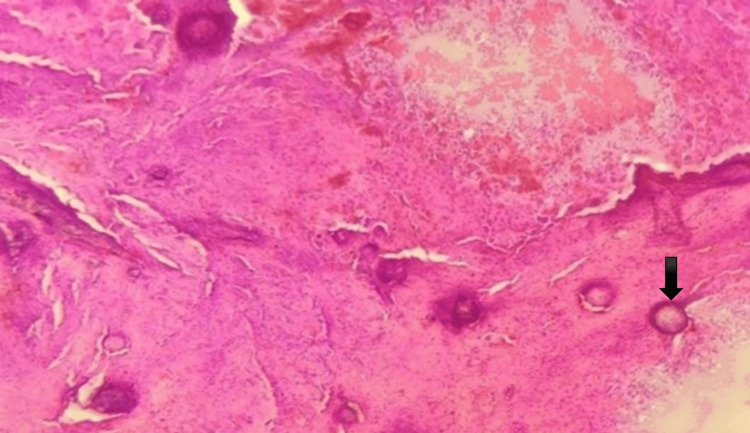
Meningioma in squash cytology showing psammoma bodies (black arrow) (10x).

Meningiomas showed typical cytological features, including whorled clusters of elongated cells and the presence of psammoma bodies. Figure 3 displays a meningioma with numerous psammoma bodies, as seen on squash smear (10x).

**Figure 4:**
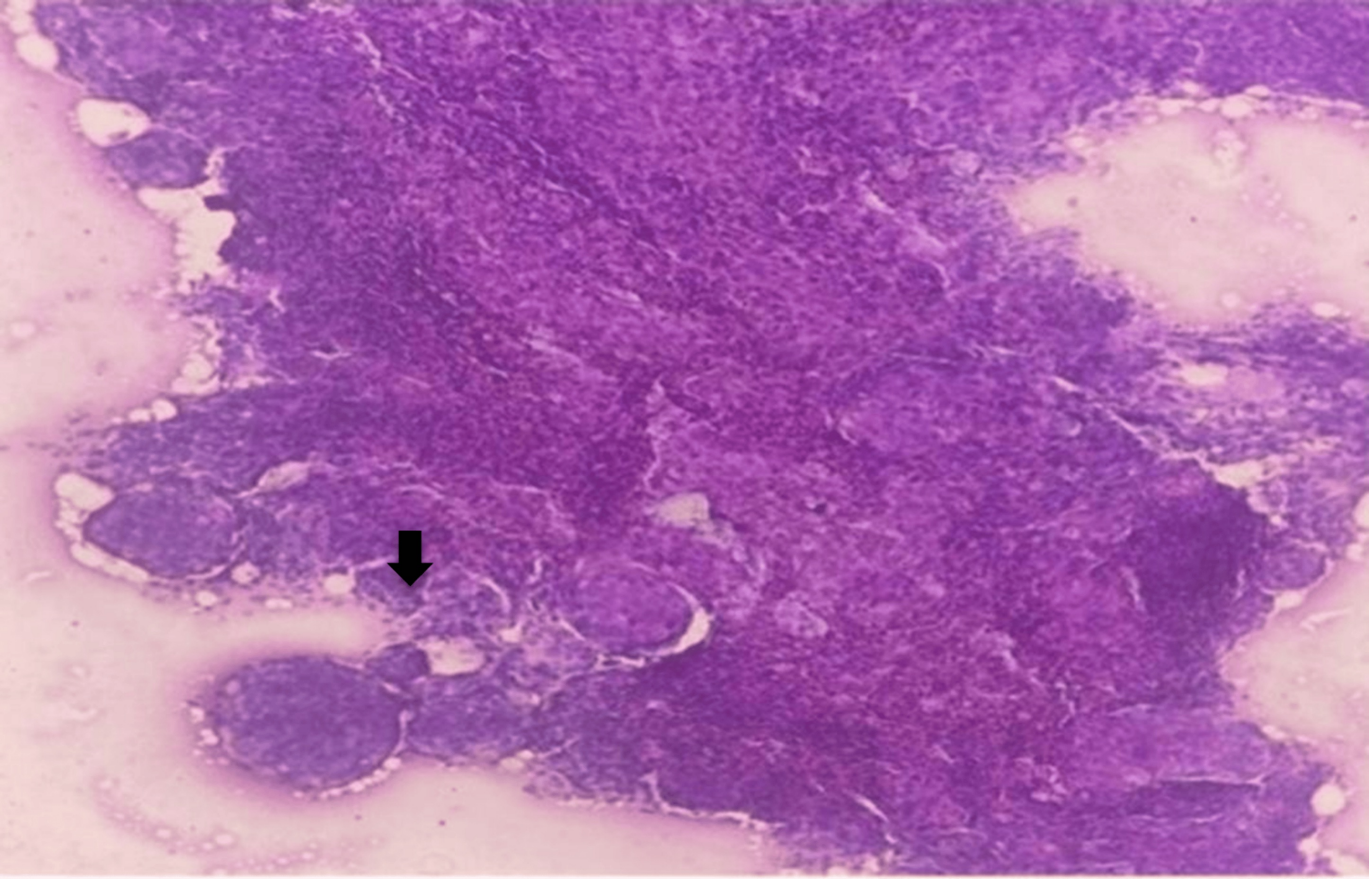
Meningothelial meningioma in squash cytology (10x) showing meningothelial whorls (black arrow).

Subtypes observed included meningothelial and transitional meningiomas, both categorized as WHO grade I. Figure 4 highlights meningothelial meningioma on squash cytology (10x), showing whorled cell arrangements typical of WHO grade I tumors.

**Figure 5:**
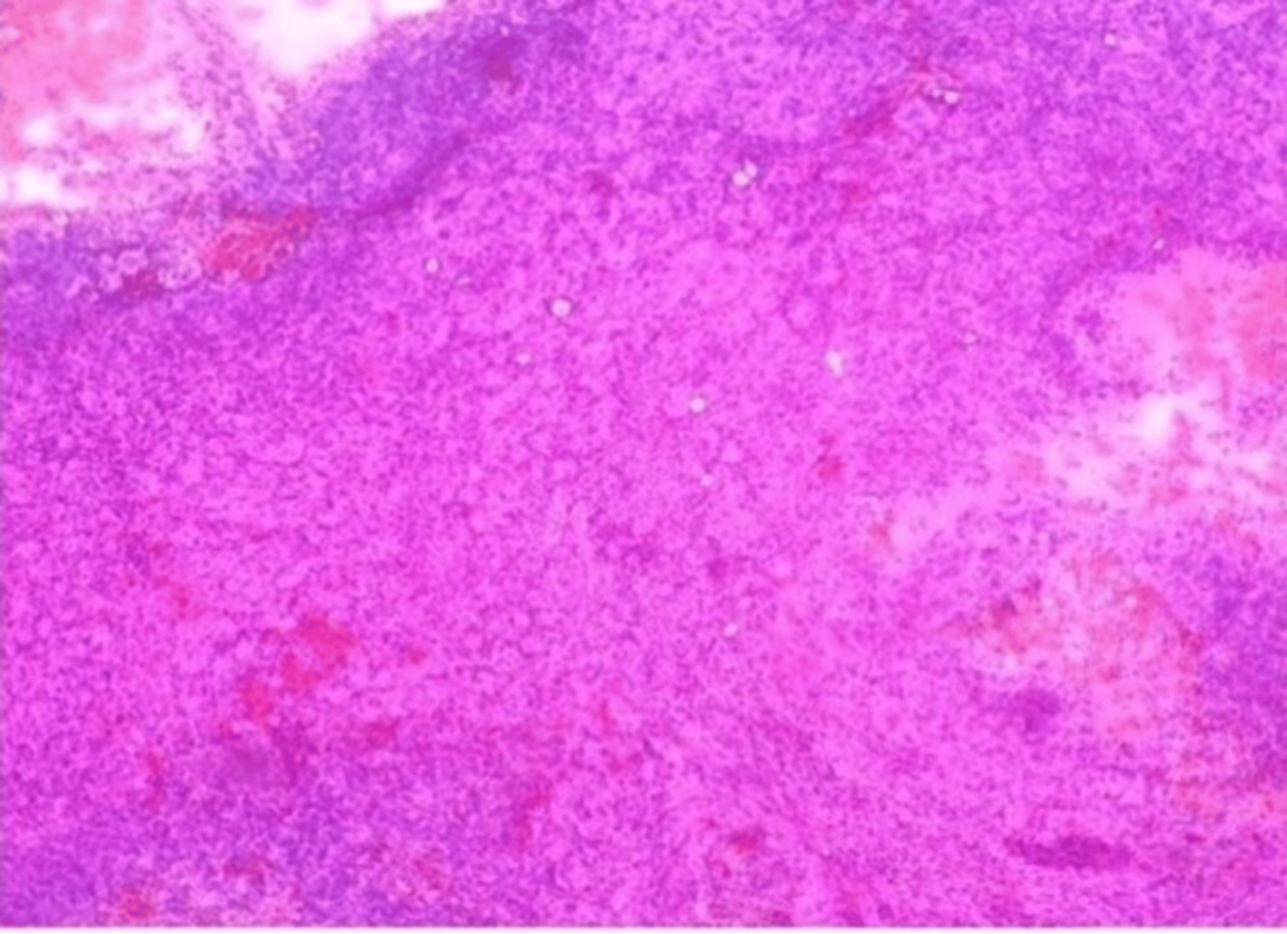
Transitional meningioma in squash cytology (10x).

Figure 5 illustrates a transitional meningioma, another WHO grade I subtype, observed on squash smear at 10x.

**Figure 6:**
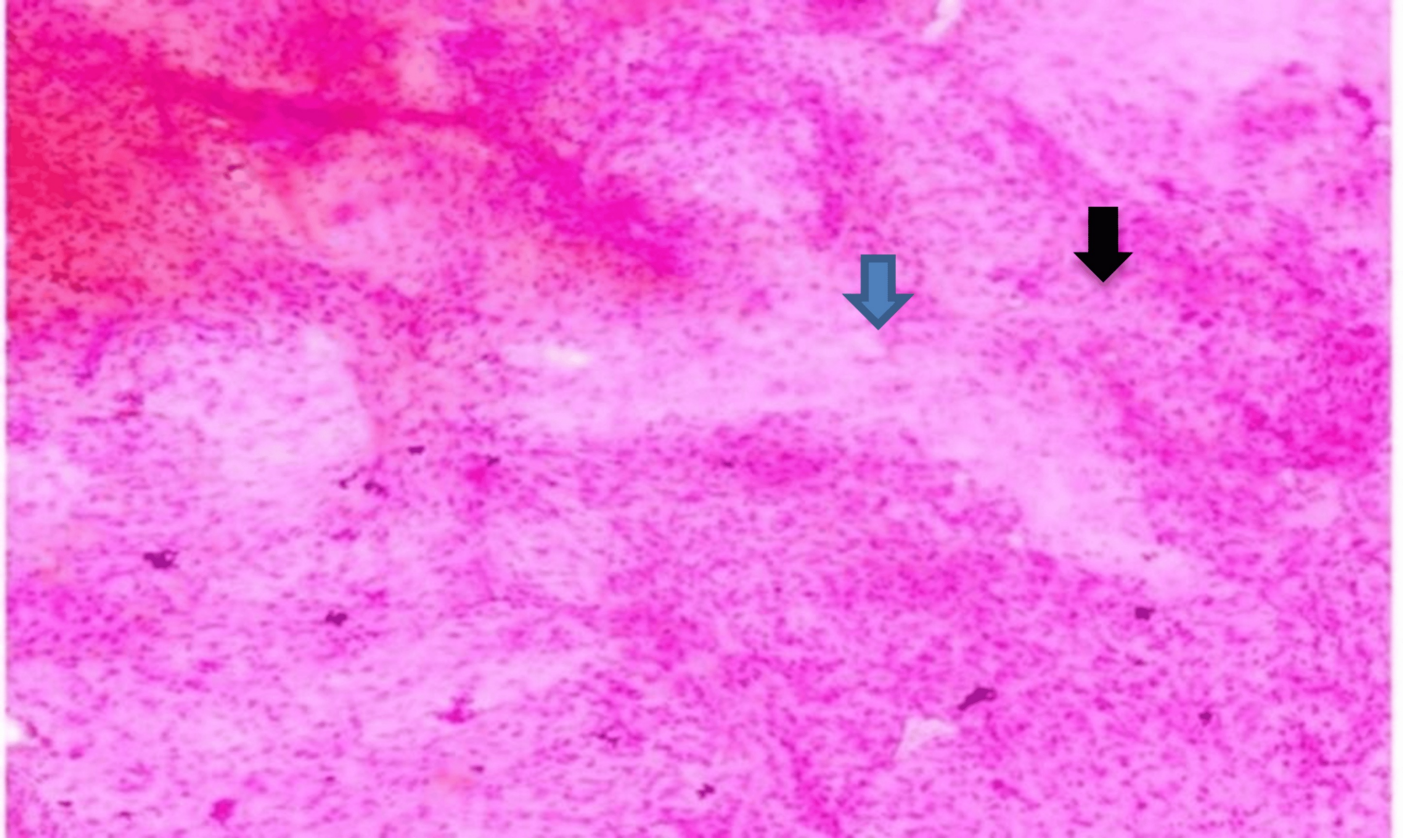
Schwannoma in squash cytology showing hypocellular (black arrow) and hypercellular areas (blue arrow) (10x).

Schwannomas were identified by their characteristic biphasic pattern of Antoni A and B areas, with spindle-shaped cells and occasional Verocay bodies. Figure 6 presents a schwannoma, demonstrating alternating hypo- and hypercellular areas in a hemorrhagic background (10x).

**Figure 7:**
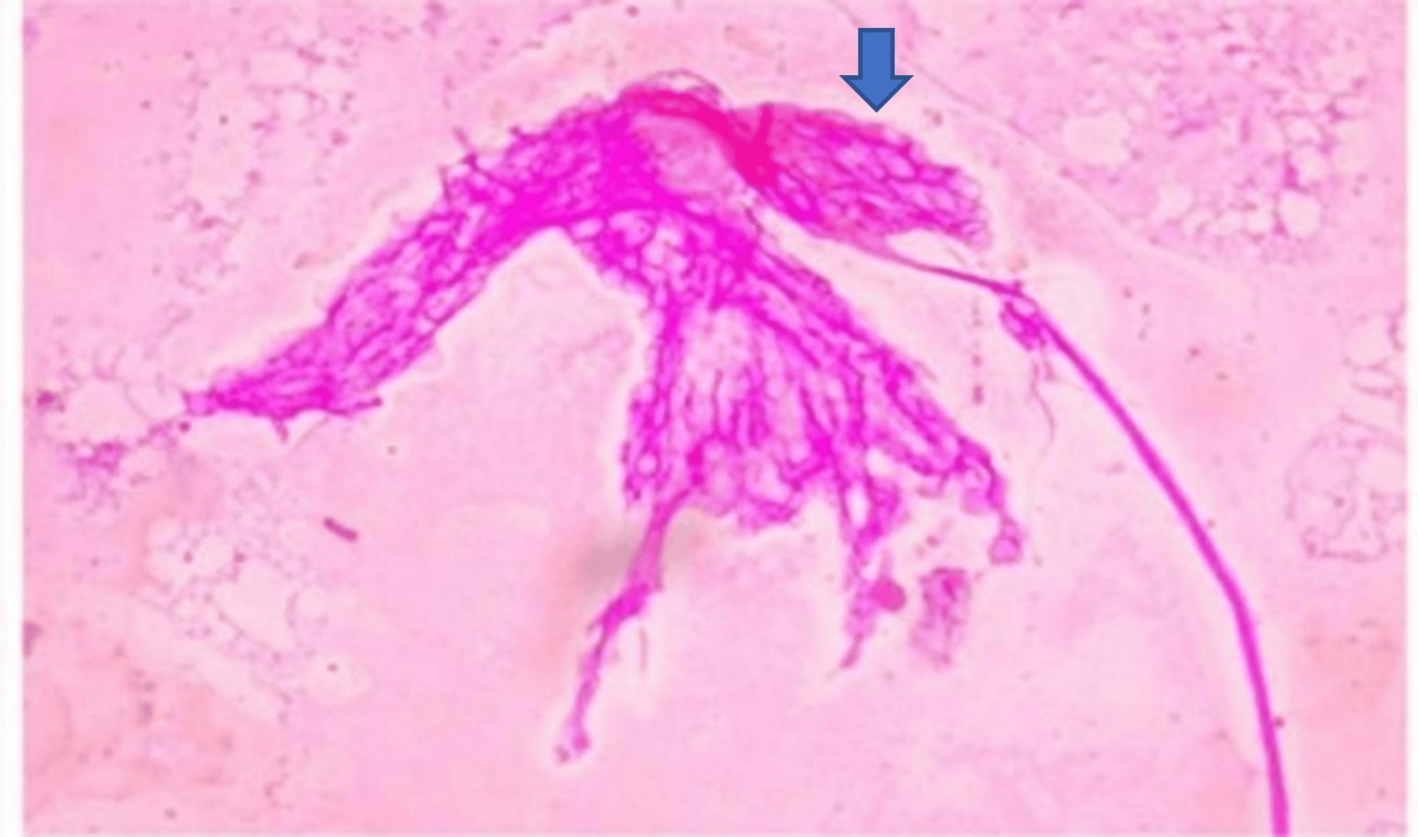
Lipoma in squash cytology showing fragments of mature adipocytes (blue arrow) (40x).

Other rare lesions included lipoma, which showed mature adipocytes, and metastatic carcinoma, which presented with clusters of malignant epithelial cells. Figure 7 shows a lipoma on squash cytology (40x), consisting of mature adipose tissue fragments.

**Figure 8:**
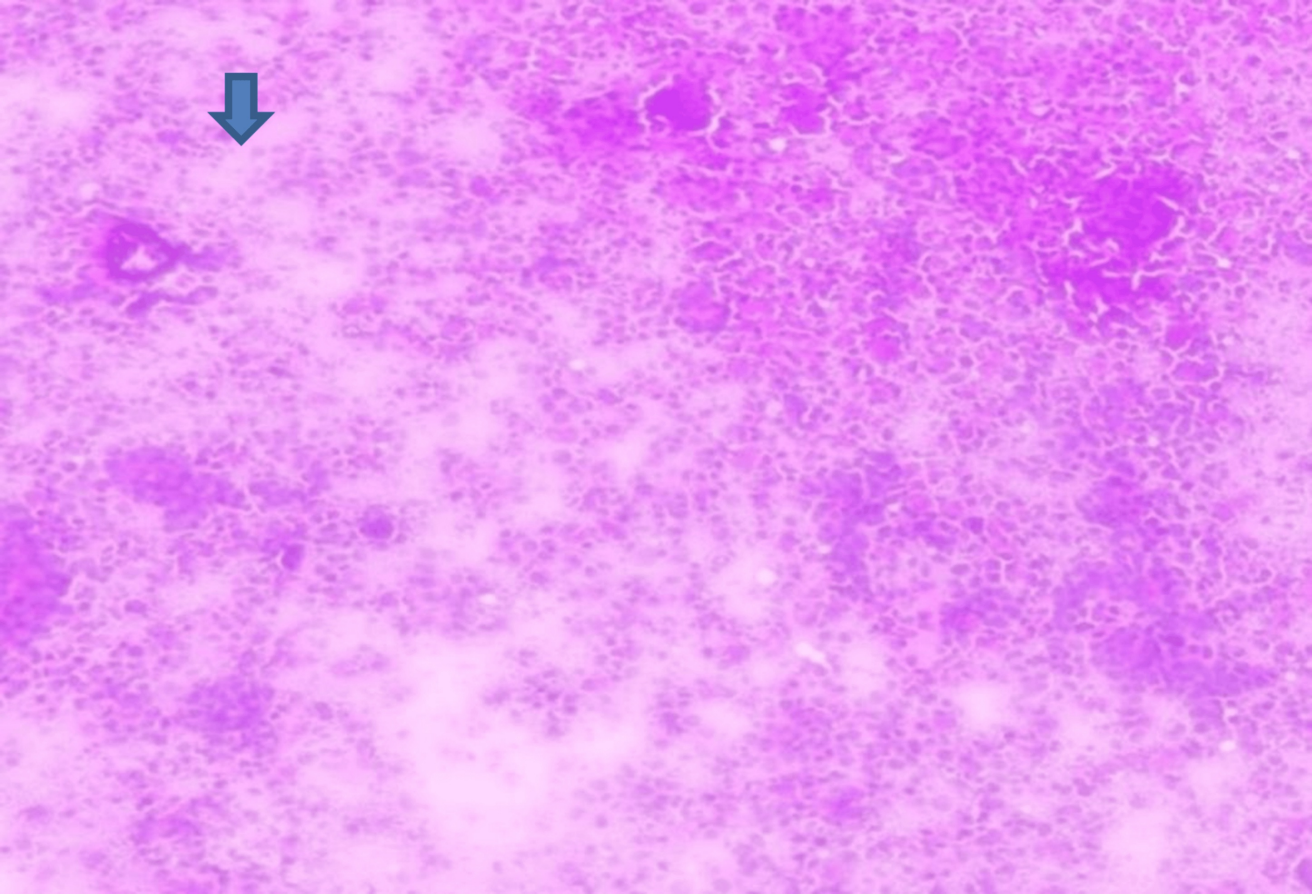
Squash cytology showing metastatic deposits in brain (blue arrow) (40x).

Figure 8 reveals metastatic carcinoma deposits in the brain, characterized by clusters of malignant epithelial cells on squash smear (40x).

**Figure 9:**
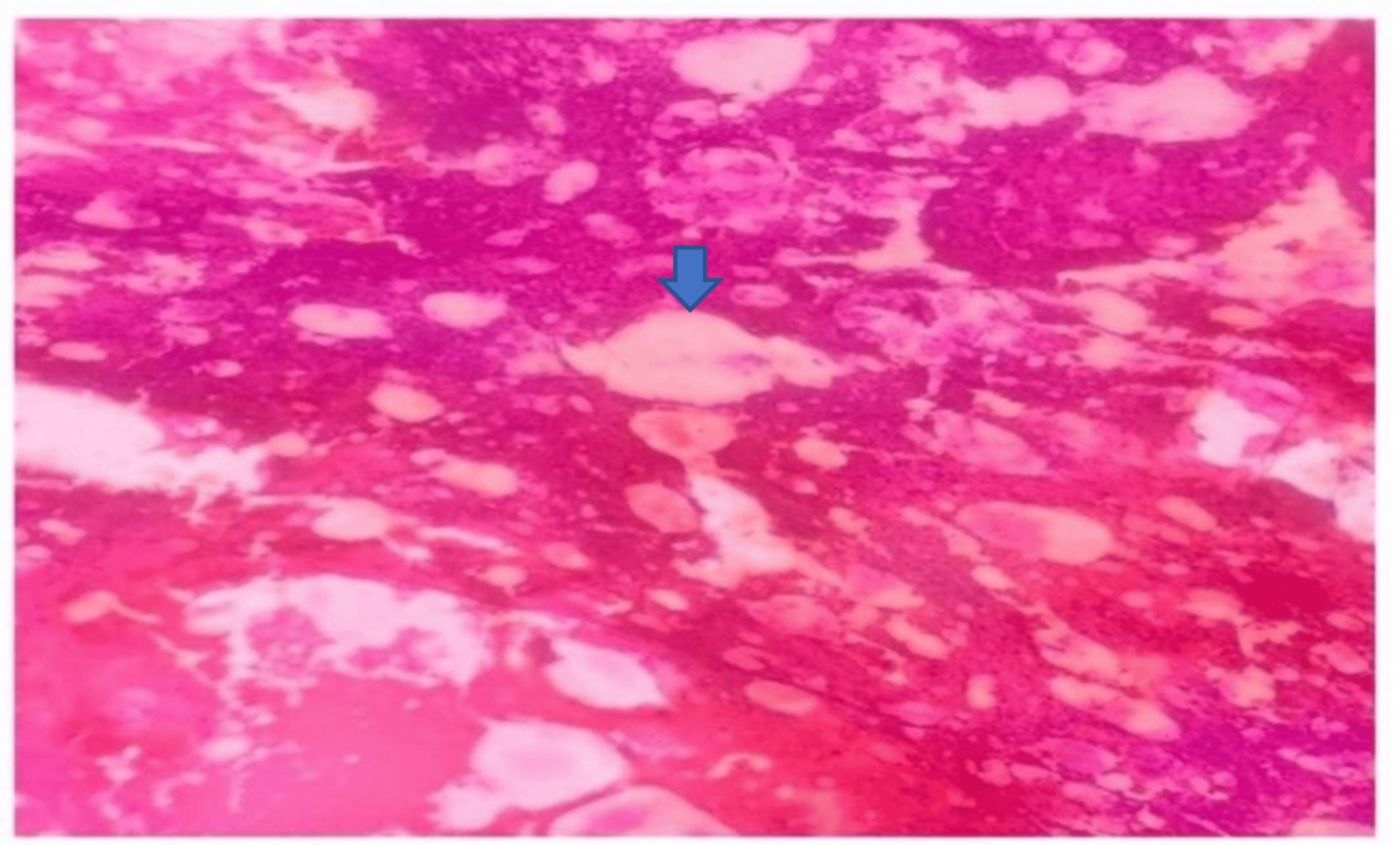
Hemangioma in squash cytology showing a hemorrhagic background with structures that resemble blood vessels (blue arrow) (40x).

Hemangiomas displayed vascular channels with erythrocyte-filled lumina in a hemorrhagic background. Figure 9 captures a hemangioma, with a prominent hemorrhagic background and vessel-like structures visible on smear (40x).

**Figure 10:**
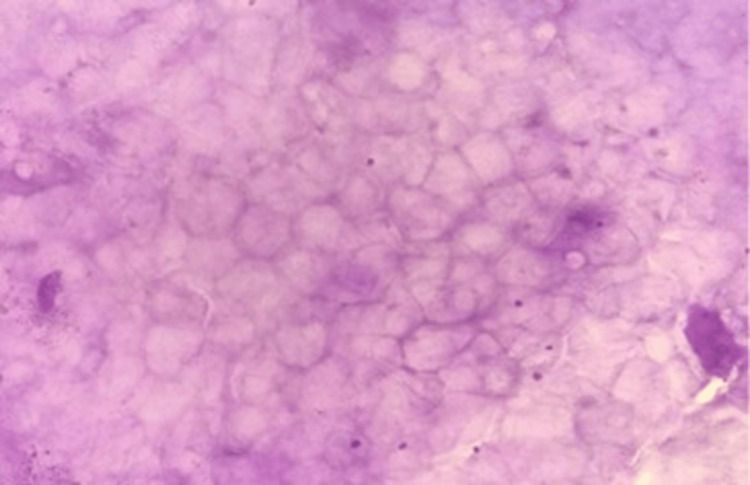
Epidermoid cyst in squash cytology (40x).

The epidermoid cyst appeared as acellular keratinous debris with anucleate squames. Figure 10 features an epidermoid cyst, with acellular keratin debris and anucleate squames, as seen under 40x magnification.

Histopathological confirmation

Histopathological examination confirmed glioma in 20 patients (40%), meningioma in 14 (28%), schwannoma in seven (14%), and metastasis in two (4%). Gliomas (40%) and meningiomas (28%) were again the most common. Additional lesions confirmed histologically included schwannomas, metastasis, lipoma, epidermoid cyst, medulloblastoma, dysembryoplastic neuroepithelial tumor (DNET), AV malformation, and an unclassified neoplastic lesion.

Rare lesions included dysembryoplastic neuroepithelial tumor (DNET), arteriovenous malformation, desmoplastic medulloblastoma, and lipoma. Histology was used as the gold standard for all diagnostic comparisons. Table 1 presents the demographic and clinical characteristics of the study population (N = 50), including age and gender distribution, tumor types, anatomical site of lesions, and diagnoses based on squash smear cytology and histopathology.

**Table 1:** Demographic and clinical characteristics of the study population.

Variable	Category	Frequency	Percent (%)
Age group	1-10 Years	2	4.0
	11-20 Years	4	8.0
	21-30 Years	7	14.0
	31-40 Years	11	22.0
	41-50 Years	13	26.0
	51-60 Years	8	16.0
	>60 Years	5	10.0
	Total	50	100.0
Gender	Male	32	64.0
	Female	18	36.0
	Total	50	100.0
Tumor type	Glioma	20	40.0
	Meningioma	15	30.0
	Schwannoma	4	8.0
	Metastasis	2	4.0
	Hemangioma	1	2.0
	Medulloblastoma	2	4.0
	Neurofibroma	2	4.0
	Vascular malformation	1	2.0
	Mesenchymal tumor	1	2.0
	Epidermoid cyst	1	2.0
	Lipoma	1	2.0
	Total	50	100.0
Site	Frontal	18	36.0
	Temporal	4	8.0
	Parietal	2	4.0
	Occipital	1	2.0
	Cerebello-pontine angle	5	10.0
	Brain stem	1	2.0
	Cerebellum	4	8.0
	Ventricle	4	8.0
	Base of skull	2	4.0
	Spine	7	14.0
	Insular cortex	1	2.0
	Multiple sites	1	2.0
	Total	50	100.0
Diagnosis	Glioma	20	40.0
	Meningioma	16	32.0
	Schwannoma	6	12.0
	Metastasis	2	4.0
	Hemangioma	2	4.0
	Epidermoid cyst	1	2.0
	Small round cell tumor	1	2.0
	Lipoma	1	2.0
	Spindle cell lesion	1	2.0
	Total	50	100.0
Diagnosis	Glioma	20	40.0
	Meningioma	14	28.0
	Schwannoma	7	14.0
	Metastasis	2	4.0
	Hemangioma	1	2.0
	Lipoma	1	2.0
	Epidermoid cyst	1	2.0
	Medulloblastoma	1	2.0
	Dysembryoplastic NT (DNET)	1	2.0
	AV malformation	1	2.0
	Others (unclassified neoplastic lesion)	1	2.0
	Total	50	100.0

Diagnostic agreement across modalities

Diagnostic agreement among radiological findings, squash smear cytology, and histopathological examination (HPE) was assessed. In 76% of cases (n = 38), all three modalities: radiology, cytology, and histopathology, provided matching diagnoses. In five cases (10%), the cytological diagnosis matched the final histopathological diagnosis, while the radiological findings differed. Only one case (2%) showed a complete mismatch among all three modalities. Table 2 reveals the diagnostic concordance among radiological findings (A), squash smear cytology (B), and histopathological diagnosis (C). In 76% of cases, all three modalities provided the same diagnosis, while varying degrees of discordance were observed in the remaining cases.

**Table 2:** Comparison between radiological diagnosis (A), squash smear cytology (B), and histopathological diagnosis (C).

Diagnostic concordance	Frequency	Percent (%)
A, B, and C reveal the same diagnosis	38	76.0
A and C reveal the same diagnosis, but B is different	2	4.0
B and C reveal the same diagnosis, but A is different	5	10.0
A and B reveal the same diagnosis, but C is different	4	8.0
A, B, and C are all different	1	2.0
Total	50	100.0

Diagnostic performance analysis demonstrated a high level of agreement between squash smear cytology and histopathological examination, with cytology showing higher diagnostic accuracy than radiological evaluation. Sensitivity, specificity, positive predictive value (PPV), and negative predictive value (NPV) were calculated to validate the accuracy of cytological diagnoses using histopathology as the gold standard. The association between squash smear findings and histopathological results was statistically significant (p = 0.001). Table 3 displays the correlation matrix between the three diagnostic modalities. The correlation coefficient between squash smear cytology and histopathology was highest (r = 0.991), indicating strong diagnostic alignment.

**Table 3:** Correlation between HPE, radiological diagnosis, and squash smear diagnosis. HPE: histopathological examination.

Variables	HPE diagnosis	Radiological diagnosis	Squash smear diagnosis
HPE diagnosis	1	0.980760318	0.991779176
Radiological diagnosis	0.980760318	1	0.98310795
Squash smear diagnosis	0.991779176	0.98310795	1

Diagnostic concordance

Out of 50 cases, complete diagnostic concordance between squash smear cytology and histopathology, including both tumor type and WHO grade, was observed in 38 cases (76%). When partial concordance was included, where the tumor category was correctly identified but there were differences in subtype or grade, the diagnostic accuracy increased to 92% (46 cases). The highest concordance was observed in low-grade gliomas and meningiomas. Most of the discordant cases involved minor grading discrepancies, particularly between WHO grade II and grade III gliomas.

Lesion-wise diagnostic accuracy was highest for schwannomas, metastases, epidermoid cysts, and lipomas (100%). Accuracy for meningiomas was 93.7%, and for gliomas, 90%. Hemangiomas and a few rare tumor types were more likely to be misclassified. Table 4 shows the lesion-wise diagnostic accuracy of squash smear cytology. It achieved 100% accuracy for schwannoma, metastasis, lipoma, and epidermoid cyst. Meningiomas had 93.7% accuracy, while gliomas had 90%. Hemangioma and two rare lesions (spindle cell and small round cell tumors) had lower accuracy.

**Table 4:** Diagnostic accuracy of squash smear cytology.

Diagnosis by squash smear cytology	Misdiagnosed cases	Correctly diagnosed	Diagnostic accuracy
Gliomas (20)	2	18	90%
Meningioma (16)	1	15	93.7%
Schwannoma (6)	Nil	6	100%
Brain metastasis (2)	Nil	2	100%
Hemangioma (2)	1	1	50%
Epidermoid cyst (1)	Nil	1	100%
Lipoma (1)	Nil	1	100%
Spindle cell lesion (1)	1	-	-
Small round cell tumor (1)	1	-	-

## Discussion

It is demonstrated in this study that squash smear cytology is very useful for intraoperative diagnosis of CNS-SOLs, as it agrees strongly with histopathology and offers many benefits over radiological imaging for surgeons during surgery. Squash smear cytology was found to be very accurate (86%) and reliable, especially in resource-limited settings where frozen section facilities are not available. This has been highlighted in prior studies emphasizing its utility as a rapid, cost-effective alternative for intraoperative diagnosis [12]. In our study, gliomas were the most commonly found tumors in all three methods: radiology, cytology, and histopathology, which is in line with their common occurrence in CNS neoplasms [6]. Meningiomas and schwannomas were the next most common types. The pattern is similar to what has been found in previous studies that used squash smear to assess CNS tumors [13]. Schwannomas, metastases, epidermoid cysts, and lipomas were the lesions where the diagnostic accuracy was highest, reaching 100%. In 93.7% of cases, meningiomas were correctly found, and gliomas were correctly found in 90% of cases.

Squash cytology showed a high sensitivity of 95% and a perfect specificity of 100%, but the negative predictive value was only 67.7%. The result means that a positive test is very helpful for diagnosing cancer, but a negative test cannot always rule out the possibility. When the number of cells is low, the tumor has died, or the sample comes from an unrepresentative area, false negatives may occur. Therefore, depending only on squash smear during surgery might miss certain cases, so it is important to interpret the results with other information in mind.

To provide a more detailed evaluation of diagnostic performance, accuracy was assessed using both complete and partial concordance metrics. Diagnostic accuracy by complete concordance (DACC), defined as an exact match between cytological and histopathological diagnoses, including both tumor type and WHO grade, was observed in 76% of cases (n = 38). When partial concordance was also considered, where the broad tumor category was correctly identified but there were minor discrepancies in histological subtype or WHO grade, the diagnostic accuracy after applying partial concordance (DAPC) increased to 92% (n = 46) [14]. Concordance across WHO grades was highest in low-grade gliomas and meningiomas. Minor disagreements in tumor grading, particularly between WHO grade II and grade III gliomas, accounted for most of the partially concordant cases. These findings underscore the high diagnostic reliability of squash smear cytology, even in settings where comprehensive intraoperative classification may be limited [15]. Table 5 compares diagnostic modalities (squash smear cytology, frozen section, imprint cytology, histopathology, and radiological imaging) in terms of accuracy, turnaround time, and usability. Squash smear cytology had 86% diagnostic accuracy with a rapid turnaround time of 10-15 minutes.

**Table 5:** Comparison of diagnostic modalities for intraoperative CNS tumor evaluation. CNS: central nervous system.

Modality	Diagnostic accuracy	Turnaround time	Comments
Squash smear cytology	86%	~10–15 minutes	Rapid and cost-effective
Frozen section	90%	~20–30 minutes	Requires cryostat; limited in soft tumors
Imprint cytology	78%	~10 minutes	Useful when tissue is minimal
Histopathology (gold standard)	100%	Several days	Required for final diagnosis
Radiological imaging	82%	Variable	Non-invasive; limited specificity

In a few cases, the results from cytology and histopathology were not the same. In one instance, a glioma was found cytologically, but the final diagnosis was dysembryoplastic neuroepithelial tumor (DNET) after histological examination. The reason for the misclassification is likely that low-grade gliomas and rare mixed neuronal-glial tumors share many features [15]. A case that was thought to be glioma by imaging and reported as such cytologically was later confirmed by histology to be metastatic carcinoma. Such cases point out that cytology can be limited when looking at tumors with unique cell types or when the smears are not very cellular or damaged. The cells in reactive gliosis, low-grade gliomas, and some secondary tumors can look very similar, which could lead to mistakes in diagnosis [16]. Even so, squash smear cytology is very useful in practice. It gives results in 10 to 15 minutes, uses only a small amount of tissue, and does not produce the typical artifacts seen in frozen sections, especially in delicate CNS tissues [17]. It has been found in previous research that squash smears confirm the presence of tumors and also help surgeons judge the quality of tissue during stereotactic biopsies, so that extra samples can be taken if necessary during surgery [18].

Even though the findings look positive, this study does have some limitations. The study was performed at just one center and included 50 patients. All cytological interpretations were performed by a single experienced cytopathologist. While this ensured consistency in reporting, the absence of multiple independent observers may limit the generalizability of the findings, as inter-observer variability could not be assessed. There may be fewer rare lesions and borderline cases in the data. In addition, the study did not use immunohistochemistry or molecular diagnostics, which are now crucial for tumor grading and classification according to the WHO [19]. The study points out that squash smear cytology is still valuable for making decisions during surgery. Because it can accurately tell neoplastic from non-neoplastic lesions and describe common tumor types, it is a suitable option when rapid histology is required and frozen sectioning cannot be done. Yet, cytological findings should always be considered in the context of imaging and the patient's medical history, and histopathology should be done when possible. Squash smear cytology is still an important method used in neurosurgical pathology. If done by skilled staff and considered together with other medical fields, it can help ensure accurate and prompt diagnoses and better results from surgery. Research in the future should involve larger groups, include data from different centers, and investigate the use of cytology together with digital pathology and artificial intelligence to improve the accuracy of diagnosis [20].

## Conclusions

Squash smear cytology is a precise and technically demanding diagnostic technique for evaluating CNS lesions intraoperatively. It requires careful tissue handling, optimal smear preparation, and experienced interpretation to distinguish between various tumor types and grades, especially due to the cellular complexity of central nervous system tumors. The technique is quick, cheap, requires only a small tissue sample, and provides clear specifics of cellular structure. Thus, squash smear cytology is an ideal diagnostic tool, especially in developing countries like India, where the price of cryostat machines is high. Cryostat is different from squash cytology, which does not need electricity for slide preparation and does not require specialized equipment and trained technicians. Freezing artifacts associated with the cryostat can also cause diagnostic difficulties. In addition, squash smear cytology should not be used as the only diagnostic modality, as the gold standard is histopathology. Squash cytology provides rapid diagnostic results and helps to assess tissue adequacy, as the use of stereotactic biopsies is increasing. In this study, squash smear cytology and histopathological evaluations were performed by two senior pathologists, each with over 10 years of experience in neuropathology. The same pathologist who evaluated the intraoperative squash smears also reported the final histopathological diagnosis for each case, ensuring consistency in interpretation. When performed by experienced specialists, squash smear cytology serves as a reliable and efficient method for the rapid intraoperative diagnosis of CNS lesions.
